# Chaos and Coexisting Attractors of Kolmogorov-Type Permanent-Magnet Synchronous Generators

**DOI:** 10.3390/e28050512

**Published:** 2026-05-01

**Authors:** Dongdong Wang

**Affiliations:** Department of Electrical Engineering and Automation, School of Electrical and Information Engineering, Hunan Institute of Technology, Hengyang 421002, China; wangdongdongbox@126.com

**Keywords:** bifurcation, chaos, permanent-magnet synchronous generator, coexisting attractor

## Abstract

This paper investigates the dynamic behavior of a Kolmogorov-type permanent-magnet synchronous generator for wind power systems. Firstly, the chaotic model of the salient-pole permanent-magnet synchronous generator is derived and subsequently transformed into a Kolmogorov-type system. Secondly, by analyzing the derived Kolmogorov system, the system’s stability is established, and the boundary ellipsoid of the chaotic attractor is determined via the Casimir energy function. Thirdly, the analysis focuses on the mechanisms leading to chaos, including period-doubling bifurcation and the onset of double Hopf bifurcation. Finally, the basins of attraction associated with the coexisting static attractors are determined to characterize their long-term dynamical behavior. The analytical results show good agreement with the numerical simulations.

## 1. Introduction

To address the environmental crisis posed by global warming, renewable energy technologies have been vigorously developed. As a crucial clean energy solution, wind energy systems harness wind power to generate electricity, in which the direct-drive permanent-magnet synchronous generator (PMSG) is widely applied. By eliminating the gearbox, installation and maintenance costs are significantly reduced [1].

The Lorenz equations serve as a fundamental framework for analyzing complex nonlinear dynamics in electrical machines, such as permanent-magnet synchronous motors (PMSMs) and switched reluctance motors [2,3]. Various control strategies are proposed to suppress the nonlinear oscillations in the PMSM [4]. Similarly, the PMSG employed in wind power systems exhibits irregular behaviors that significantly compromise its service time and operational safety. Frequency-domain analysis is primarily employed to investigate the stability and nonlinear behaviors of the non-salient pole PMSG, i.e., small signal analysis [5,6,7,8] in which characteristic root traces reveal the effects of the operating parameters. A mathematical model of the fractional-order PMSG is formulated, facilitating the numerical analysis of chaotic pathways at different orders [9]. Furthermore, a variety of methods are proposed to stabilize the non-salient pole PMSG such as neural dynamics control [10], adaptive control [11], sliding mode control [12], Lyapunov-based model predictive control [13] etc. In wind power systems, multiple PMSGs are often interconnected to enhance the overall power generation capacity. So, synchronization control is essential for PMSGs to ensure the stability of each individual unit [14,15].

Besides the Lyapunov function, Kolmogorov theory is a vital tool for stability analysis. Kolmogorov-type systems provide an effective framework for revealing chaotic attractors based on the energy cycle [16,17,18]. Casimir energy serves as a vital metric for analyzing the dynamical behaviors of the complex system [19]. While standard Lyapunov stability analysis is applicable to a wide range of dynamical systems, Casimir energy theory offers an effective means of estimating the chaotic boundary in Kolmogorov-type systems. The utility of each method is contingent upon the specific application. Moreover, given that stable equilibrium points can coexist under specific conditions, initial values of the state variables play a pivotal role in revealing hidden coexisting attractors [20,21].

In non-salient PMSG, extensive research has been conducted to analyze its nonlinear behaviors, i.e., Hopf bifurcation and chaotic behavior [22,23]. However, considering their significant influence on the rated state and design parameters, the coexisting attractors and the boundary characterization of the chaotic attractors have not been thoroughly investigated. Furthermore, owing to the inherent disparity in stator inductance, the salient-pole PMSG exhibits significantly more pronounced nonlinearities than its non-salient counterpart. Without loss of generality, the mathematical model of the salient-pole PMSG will be developed and analyzed in the following sections. In Section 2, viewing the system from an energy perspective, the salient-pole PMSG model is recast into the framework of a Kolmogorov system. In Section 3, the energy function is employed to investigate the PMSG stability and delineate the boundary ellipsoid of the chaotic attractor. In Section 4, the stability characteristics of the salient-pole Kolmogorov system are analyzed. In Section 5, the mechanism underlying the coexisting stable attractors and period-doubling bifurcation is examined. In Section 6, the double Hopf bifurcation and the basins of attraction are investigated. Section 7 concludes this paper.

## 2. Kolmogorov Model of the Salient-Pole PMSG

**Figure 1:**
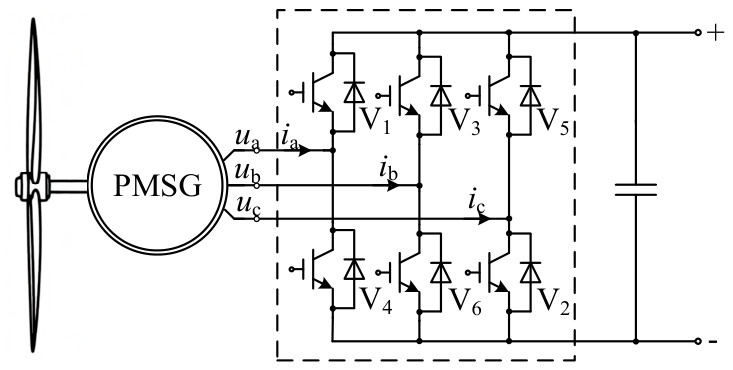
Schematic diagram of the salient-pole PMSG.

The schematic diagram of the salient-pole PMSG is depicted in Figure 1 where V*_i_* (*i* = 1, 2, …, 6) is the switch of the three-phase voltage source rectifier; *i*_a_, *i*_b_ and *i*_c_ are the input currents of the rectifier; *u*_a_, *u*_b_ and *u*_c_ are the phase voltages controlled by the generator-side rectifier.

Following the generator-reference convention [1], the mathematical model for the salient-pole PMSG is formulated as follows:

(1) $$\left\{\begin{array}{l} \frac{di_{d}}{dt}=-\frac{R}{L_{d}}i_{d}+\frac{L_{q}}{L_{d}}\omega_{e}i_{q}-\frac{1}{L_{d}}u_{d}\\ \frac{di_{q}}{dt}=-\frac{R}{L_{q}}i_{q}-\frac{L_{d}}{L_{q}}\omega_{e}i_{d}+\frac{\psi_{f}}{L_{q}}\omega_{e}-\frac{1}{L_{q}}u_{q}\\ \frac{d\omega_{e}}{dt}=\frac{n_{p}}{J}T_{m}-\frac{n_{p}}{J}[\frac{3}{2}n_{p}(\psi_{f}i_{q}-(L_{d}-L_{q})i_{d}i_{q})]-\frac{B}{J}\omega_{e} \end{array}\right.$$

where *i*_d_, *i*_q_ and *ω*_e_ correspond to the d- and q-axis components of the stator currents and the electrical angle of the rotor, respectively; *ψ*_f_ and *T*_m_ represent the flux amplitude of the rotor and the mechanical torque, respectively; *R*, *n*_p_, *J* and *B* denote the stator resistance per phase, the number of pole pairs, the moment of inertia and the viscous friction coefficient, respectively; *u*_d_ and *u*_q_ are the d- and q-axis components of the stator voltages *u*_a_, *u*_b_ and *u*_c_; *L*_d_ and *L*_q_ are the d- and q-axis inductances of the stator, respectively; $T_{m}=\frac{1}{2}\rho \pi r^{3}v^{2}C_{Q}$, where *ρ*, *r*, *v* and *C*_Q_ denote the air density, the radius of the turbine, the wind speed and the torque coefficient, respectively; *T*_m_ is constant with the fixed wind speed *v*.

For convenience of analysis, model (1) can be further reduced via a time-scaling transformation $t=\tau \tilde{t}$ and an affine transformation $x=\xi \tilde{x}$, where $x={\left[i_{d}\;\;i_{q}\;\;\omega_{e}\right]}^{T}$, $\tilde{x}={\left[x\;\;y\;\;z\right]}^{T}$, $\xi =\left[\begin{array}{l} \xi_{d}\;\;\;\;0\;\;\;\;\;\;0\\ 0\;\;\;\;\;\xi_{q}\;\;\;\;\;0\\ 0\;\;\;\;\;\;0\;\;\;\;\xi_{\omega_{e}}\; \end{array}\right]=\left[\begin{array}{l} bk\;\;\;\;0\;\;\;\;0\\ 0\;\;\;\;\;\;k\;\;\;\;0\\ 0\;\;\;\;\;\;0\;\;\;\;1/\tau \end{array}\right]$, $b=\frac{L_{q}}{L_{d}}$, $k=\frac{2}{3}\frac{B}{{n_{p}}^{2}\tau \psi_{f}}$ and $\tau =\frac{L_{q}}{R}$.

Then, the model of the salient-pole PMSG becomes

(2) $$\left\{\begin{array}{l} \frac{dx}{d\tilde{t}}=-bx+zy-{\tilde{u}}_{d}\\ \frac{dy}{d\tilde{t}}=-y-zx+\gamma z-{\tilde{u}}_{q}\\ \frac{dz}{d\tilde{t}}={\tilde{T}}_{m}-\sigma (y+z)+\varepsilon xy \end{array}\right.$$

where $\gamma =\frac{\psi_{f}}{kL_{q}}$, ${\tilde{u}}_{d}=\frac{1}{Rk}u_{d}$, ${\tilde{u}}_{q}=\frac{1}{Rk}u_{q}$, ${\tilde{T}}_{m}=\frac{n_{p}\tau^{2}}{J}T_{m}$, $\sigma =\frac{\tau B}{J}$ and $\varepsilon =\frac{3}{2}\frac{{n_{p}}^{2}\tau^{2}(L_{d}-L_{q})bk^{2}}{J}$, respectively.

The dynamics of the salient-pole PMSG can be formulated as a dissipative-forced dynamical system:

$$\dot{x}=\{x,H\}-\Lambda x+f$$

Here, **x** = [*y*_1_, *y*_2_, *y*_3_]^T^, { , } denotes the Lie-algebraic structure [16], Λ represents a positive definite diagonal matrix and **f** stands for the external torque.

To proceed, the following transformations are utilized.

(3) $$x_{1}=\alpha x,x_{2}=y,x_{3}=\beta z$$

where *α* and *β* denote non-zero constants.

Substitution of Equation (3) into Equation (2) leads to

(4) $$\left\{\begin{array}{l} {\dot{x}}_{1}=-bx_{1}+\frac{\alpha }{\beta }x_{2}x_{3}-\alpha {\tilde{u}}_{d}\\ {\dot{x}}_{2}=-x_{2}-\frac{1}{\alpha \beta }x_{1}x_{3}+\frac{\gamma }{\beta }x_{3}-{\tilde{u}}_{q}\\ {\dot{x}}_{3}=\beta {\tilde{T}}_{m}-\beta \sigma x_{2}-\sigma x_{3}+\varepsilon \frac{\beta }{\alpha }x_{1}x_{2} \end{array}\right.$$

To ensure the skew-symmetric property of the Lie–Poisson bracket [18], the parameters *α* and *β* are determined by the following equation:

(5) $$\frac{\alpha }{\beta }-\frac{1}{\alpha \beta }+\varepsilon \frac{\beta }{\alpha }=0$$

To derive the potential energy, with transformations $y_{1}=x_{3},y_{2}=x_{2},y_{3}=x_{1}-\delta$, model (4) can be further transformed as follows:

(6) $$\left\{\begin{array}{l} {\dot{y}}_{1}=\varepsilon \frac{\beta }{\alpha }y_{3}y_{2}-\sigma y_{1}+(\varepsilon \frac{\beta \delta }{\alpha }-\beta \sigma )y_{2}+\beta {\tilde{T}}_{m}\\ {\dot{y}}_{2}=-\frac{1}{\alpha \beta }y_{3}y_{1}-y_{2}-(\frac{\delta }{\alpha \beta }-\frac{\gamma }{\beta })y_{1}-{\tilde{u}}_{q}\\ {\dot{y}}_{3}=\frac{\alpha }{\beta }y_{2}y_{1}-by_{3}-b\delta -\alpha {\tilde{u}}_{d} \end{array}\right.$$

To ensure that the skew-symmetric property of the Lie–Poisson bracket is satisfied, letting $\frac{\delta }{\alpha \beta }-\frac{\gamma }{\beta }=\varepsilon \frac{\beta \delta }{\alpha }-\beta \sigma$, we have

(7) $$\delta =\frac{-\alpha \gamma +\alpha \beta^{2}\sigma }{\varepsilon \beta^{2}-1}$$

To cast model (6) into a more compact form, by setting $e_{1}=\varepsilon \frac{\beta }{\alpha }$, $e_{2}=-\frac{1}{\alpha \beta }$, $e_{3}=\frac{\alpha }{\beta }$ and $c_{1}=\varepsilon \frac{\beta \delta }{\alpha }-\beta \sigma$, model (6) can be rearranged as

(8) $$\left\{\begin{array}{l} {\dot{y}}_{1}=e_{1}y_{2}y_{3}-\sigma y_{1}+c_{1}y_{2}+\beta {\tilde{T}}_{m}\\ {\dot{y}}_{2}=e_{2}y_{1}y_{3}-y_{2}-c_{1}y_{1}-{\tilde{u}}_{q}\\ {\dot{y}}_{3}=e_{3}y_{1}y_{2}-by_{3}-b\delta -\alpha {\tilde{u}}_{d} \end{array}\right.$$

The inverse of principal moment of inertia of model (8) is defined as

(9) $$\Pi =\mathrm{diag}\{\Pi_{1},\Pi_{2},\Pi_{3}\}=\mathrm{diag}\{1,1+e_{3},1+e_{1}+e_{3}\}$$

Consequently, the Hamiltonian function is derived as

(10) $$\begin{array}{l} H=K+U\\ =\frac{1}{2}({y_{1}}^{2}+(1+e_{3}){y_{2}}^{2}+(1+e_{1}+e_{3}){y_{3}}^{2})+c_{1}y_{3} \end{array}$$

where *K* and *U* denote the kinetic energy and the potential energy, respectively. Ultimately, model (8) reduces to a Kolmogorov system given by

(11) $$\begin{array}{ll} \dot{x} & =\{x,H\}-\Lambda x+f\\ & =x\times (\nabla K+\nabla U)-\Lambda x+f\\ & =\left(\begin{array}{c} e_{1}y_{2}y_{3}+c_{1}y_{2}\\ e_{2}y_{1}y_{3}-c_{1}y_{1}\\ e_{3}y_{1}y_{2} \end{array}\right)-\left(\begin{array}{c} \sigma y_{1}\\ y_{2}\\ by_{3} \end{array}\right)+\left(\begin{array}{c} \beta {\tilde{T}}_{m}\\ -{\tilde{u}}_{q}\\ -b\delta -\alpha {\tilde{u}}_{d} \end{array}\right) \end{array}$$

Here, *e*_1_ + *e*_2_ + *e*_3_ = 0.

$\Lambda =\left(\begin{array}{ccc} \Lambda_{1}=\sigma & 0 & 0\\ 0 & \Lambda_{2}=1 & 0\\ 0 & 0 & \Lambda_{3}=b \end{array}\right)$, $f=\left(\begin{array}{c} \beta {\tilde{T}}_{m}\\ -{\tilde{u}}_{q}\\ -b\delta -\alpha {\tilde{u}}_{d} \end{array}\right)$, $\{x,H\}=x\times \nabla H=\left(\begin{array}{c} e_{1}y_{2}y_{3}+c_{1}y_{2}\\ e_{2}y_{1}y_{3}-c_{1}y_{1}\\ e_{3}y_{1}y_{2} \end{array}\right)$. The three terms on the right-hand side of model (11) correspond to the conservative part (from kinetic and potential energy), the dissipative part, and the external force, respectively.

The equilibria of (11) are identical in number and properties to those of model (1). The relationship between the equilibria of these two systems is described by

$$y_{1}\to \beta z,y_{2}\to y,y_{3}\to \alpha x-\delta$$

**Figure 2:**
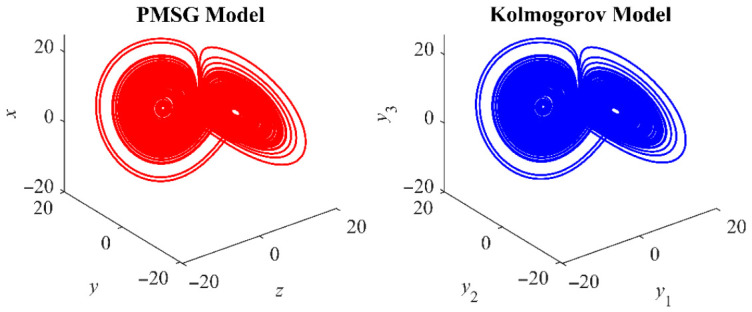
Chaotic attractor with *u*_d_ = −100 V, *u*_q_ = 0 and *T*_m_ = 0.

This transformation establishes the relationship between the PMSG model (1) and the Kolmogorov model (11). The resulting Kolmogorov-type PMSG preserves the original dynamical behaviors as shown in Figure 2. When *α* and *β* are both set to 1, the chaotic attractor of the Kolmogorov system is observed to undergo a spatial translation in the phase space upon shifting *y*_3_ by *δ*, while its shape remains identical.

Particularly, for non-salient pole PMSGs, the d-axis and q-axis inductances are equal (*L*_d_ = *L*_q_). Kolmogorov system (11) can be further formulated as follows.

(12) $$\left(\begin{array}{c} {\dot{y}}_{1}\\ {\dot{y}}_{2}\\ {\dot{y}}_{3} \end{array}\right)=\left(\begin{array}{c} c_{1}y_{2}\\ e_{2}y_{1}y_{3}-c_{1}y_{1}\\ e_{3}y_{1}y_{2} \end{array}\right)-\left(\begin{array}{c} \sigma y_{1}\\ y_{2}\\ y_{3} \end{array}\right)+\left(\begin{array}{c} \beta {\tilde{T}}_{m}\\ -{\tilde{u}}_{q}\\ -b\delta -\alpha {\tilde{u}}_{d} \end{array}\right)$$

By setting the time derivative in Equation (12) to zero, the equilibrium points are obtained as follows:

(13) $$x^{*}=\left(\begin{array}{c} {y_{1}}^{*}\\ {y_{2}}^{*}\\ {y_{3}}^{*} \end{array}\right)=\left(\begin{array}{c} \beta m\\ ({\tilde{T}}_{m}-\sigma m)/\sigma \\ \alpha (\frac{{\tilde{T}}_{m}m}{\sigma }-m^{2}-{\tilde{u}}_{d})-\delta \end{array}\right)$$

**Figure 3:**
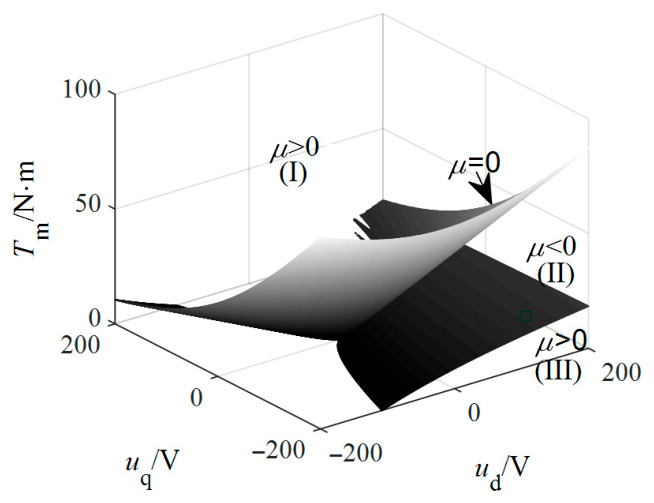
The boundary surface where *D* = 0.

Here, the three values of *m* correspond to the solutions *M*_1_, *M*_2_ and *M*_3_ of Equation (13) as [24]

(14) $$\left\{\begin{array}{ll} m_{1} & =\sqrt[{3}]{E+\sqrt{Q^{3}+E^{2}}}+\sqrt[{3}]{E-\sqrt{Q^{3}+E^{2}}}-\frac{1}{3}A_{1}\\ m_{2,3} & =-\frac{1}{2}(\sqrt[{3}]{E+\sqrt{Q^{3}+E^{2}}}+\sqrt[{3}]{E-\sqrt{Q^{3}+E^{2}}})-\frac{1}{3}A_{1}\\ & \pm \frac{1}{2}i\sqrt{3}(\sqrt[{3}]{E+\sqrt{Q^{3}+E^{2}}}-\sqrt[{3}]{E-\sqrt{Q^{3}+E^{2}}}) \end{array}\right.$$

where $A_{1}=-\frac{{\tilde{T}}_{m}}{\sigma }$, $A_{2}={\tilde{u}}_{d}+\gamma +1$, $A_{3}=-{\tilde{u}}_{q}-\frac{{\tilde{T}}_{m}}{\sigma }$, $Q=\frac{3A_{2}-{A_{1}}^{2}}{9}$ and $E=\frac{9A_{1}A_{2}-27A_{3}-2{A_{1}}^{3}}{54}$. If *D* = *Q*^3^ + *E*^2^ < 0, Equation (13) has three different real solutions; if *D* = *Q*^3^ + *E*^2^ > 0, Equation (13) has one real solution and two conjugate complex solutions; if *D* = *Q*^3^ + *E*^2^ = 0, Equation (13) has at least two equal real solutions. Based on the operating parameters in Table 1, the boundary surface of discriminant *D* is generated, as shown in Figure 3. A necessary condition for the emergence of a Lorenz-like chaotic attractor is the existence of two non-zero equilibrium points, indicating that *D* ≤ 0 in Region II. Hence, the influence of the external force **f** is of critical importance.

## 3. Chaotic Analysis of the Non-Salient Kolmogorov System

The conservative, dissipative, and external energy components of the Kolmogorov system (12) are mutually coupled, facilitating energy exchange that gives rise to complex nonlinear behaviors. An energy-based analysis of the Kolmogorov system (12) is presented below.

CASE 1:

Neglecting the second and third terms of (12), we obtain the following equation:

(15) $$\dot{x}=\{x,H\}=\left(\begin{array}{c} c_{1}y_{2}\\ e_{2}y_{1}y_{3}-c_{1}y_{1}\\ e_{3}y_{1}y_{2} \end{array}\right)$$

The volume dissipation rate of system (15) in phase space is derived as follows:

(16) $$\nabla V=\frac{\partial {\dot{y}}_{1}}{\partial y_{1}}+\frac{\partial {\dot{y}}_{2}}{\partial y_{2}}+\frac{\partial {\dot{y}}_{3}}{\partial y_{3}}=0$$

Equation (16) implies that the phase space volume *V* is conserved, as there is no dissipative mechanism to absorb energy within the system. The Casimir function, representing the internal energy of system (15), can be chosen as

(17) $$C_{e}=\frac{1}{2}<x,x>=\frac{1}{2}({y_{1}}^{2}+{y_{2}}^{2}+{y_{3}}^{2})$$

**Figure 4:**
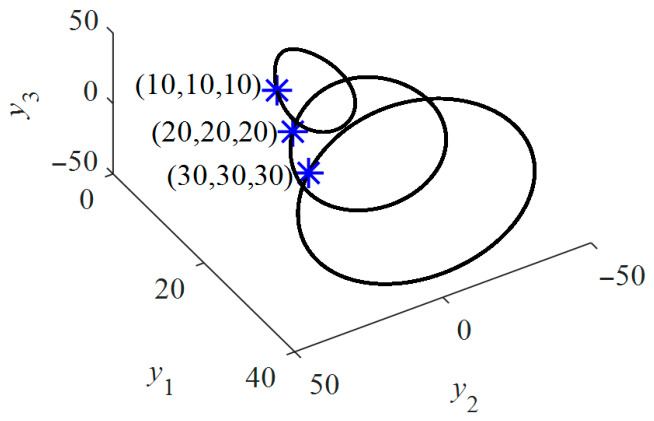
Trajectories of the state variable **x** under different initial conditions.

Here, <,> denote the inner product operator. Thus, the time derivative of the Casimir energy function ${\dot{C}}_{e}=0$ indicates that system (15) does not exchange energy with the dissipative component or the external torques. Therefore, system (15) is lossless and exhibits periodic motion, as illustrated in Figure 4.

CASE 2:

By neglecting the external torques **f** in (12), the Kolmogorov system reduces to

(18) $$\left(\begin{array}{c} {\dot{y}}_{1}\\ {\dot{y}}_{2}\\ {\dot{y}}_{3} \end{array}\right)=\left(\begin{array}{c} c_{1}y_{2}\\ e_{2}y_{1}y_{3}-c_{1}y_{1}\\ e_{3}y_{1}y_{2} \end{array}\right)-\left(\begin{array}{c} \sigma y_{1}\\ y_{2}\\ y_{3} \end{array}\right)$$

Taking the time derivative of the Casimir function (17) yields

(19) $${\dot{C}}_{e}=<\nabla C_{e},\dot{x}>=-(\sigma {y_{1}}^{2}+{y_{2}}^{2}+{y_{3}}^{2})<0$$

**Figure 5:**
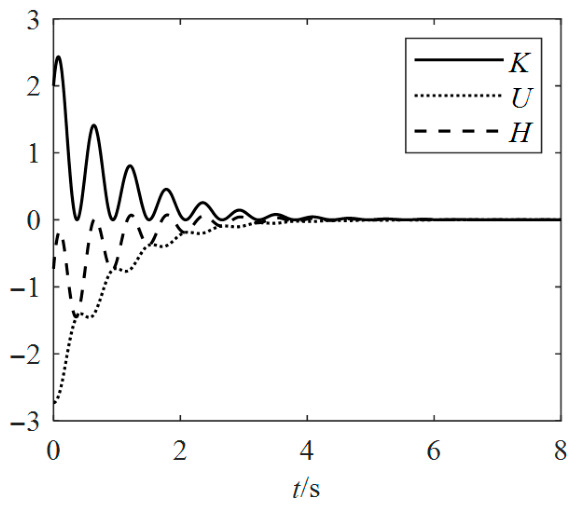
Hamiltonian energy.

Therefore, the Kolmogorov system (12) is asymptotically stable. The dissipative energy compensates for the internal energy associated with kinetic and potential energy, which confirms the principle of energy conservation. Consequently, the emergence of a chaotic attractor is precluded in this scenario. Since system (18) possesses a stable equilibrium point x* = (0, 0, 0), the Hamiltonian energy *H* is gradually dissipated by the dissipative term over time, as illustrated in Figure 5.

CASE 3:

Subject to the external torque **f**, system (11) takes the form

(20) $$\dot{x}=\{x,K\}+\{x,U\}-\nabla D_{p}+\nabla G_{p}$$

where the dissipative power is obtained as

$$D_{p}=\frac{1}{2}<x,\Lambda x>=\frac{1}{2}\sum_{i=1}^{3}\Lambda_{i}y_{i}=\frac{1}{2}(\sigma {y_{1}}^{2}+{y_{2}}^{2}+b{y_{3}}^{2})$$

and the external power is given by

$$G_{p}=<x,f>=\sum_{i=1}^{3}y_{i}f_{i}=\beta {\tilde{T}}_{m}y_{1}-{\tilde{u}}_{q}y_{2}-(b\delta +\alpha {\tilde{u}}_{d})y_{3}$$

By letting the Casimir function as

$$C_{e}=\frac{1}{2}<x,x>=\frac{1}{2}({y_{1}}^{2}+{y_{2}}^{2}+{y_{3}}^{2})$$

and {*C*_e_, *H*} = 0 [17], the time derivative of the Casimir function becomes

(21) $$\begin{array}{cc} {\dot{C}}_{e} & =<\nabla C_{e},\dot{x}>=-<x,\Lambda x>+<x,f>=-2D_{p}+G_{p}\\ & =-(\sigma {y_{1}}^{2}+{y_{2}}^{2}+b{y_{3}}^{2})+\beta {\tilde{T}}_{m}y_{1}-{\tilde{u}}_{q}y_{2}-(b\delta +\alpha {\tilde{u}}_{d})y_{3} \end{array}$$

The interplay among internal energy, external forcing and dissipative dissipation induces alternating phases of divergence and convergence in the derivative of *C*_e_, thereby giving rise to a chaotic attractor.

Moreover, by incorporating the following inequalities into Equation (21):

$$y_{1}\beta {\tilde{T}}_{m}\leq \frac{1}{2}(2\sigma -\xi ){y_{1}}^{2}+\frac{1}{2}\frac{1}{2\sigma -\xi }{\left(\beta {\tilde{T}}_{m}\right)}^{2}\;-y_{2}{\tilde{u}}_{q}\leq \frac{1}{2}(2-\xi ){y_{2}}^{2}+\frac{1}{2}\frac{1}{2-\xi }{{\tilde{u}}_{q}}^{2}\;-y_{3}(b\delta +\alpha {\tilde{u}}_{d})\leq \frac{1}{2}(2b-\xi ){y_{3}}^{2}+\frac{1}{2}\frac{1}{2b-\xi }{\left(b\delta +\alpha {\tilde{u}}_{d}\right)}^{2}$$

we obtain

$${\dot{C}}_{e}(t)\leq -\xi C_{e}(t)+\frac{1}{2}(\frac{1}{2\sigma -\xi }{\left(\beta {\tilde{T}}_{m}\right)}^{2}+\frac{1}{2-\xi }{{\tilde{u}}_{q}}^{2}+\frac{1}{2b-\xi }{\left(b\delta +\alpha {\tilde{u}}_{d}\right)}^{2})$$

Here, the parameter *ξ* serves as a weighting factor employed to modulate the spatial extent of the boundary ellipsoid.

So,

$$C_{e}(t)\leq e^{-\xi t}(C_{0}+\int_{0}^{t}e^{\xi \tau }\frac{1}{2}(\frac{1}{2\sigma -\xi }{\left(\beta {\tilde{T}}_{m}\right)}^{2}+\frac{1}{2-\xi }{{\tilde{u}}_{q}}^{2}+\frac{1}{2b-\xi }{\left(b\delta +\alpha {\tilde{u}}_{d}\right)}^{2})d\tau$$

where *C*_0_ is constant. When t→∞, the Casimir function becomes

$$C_{e}(t)\leq \frac{1}{2\xi }(\frac{1}{2\sigma -\xi }{\left(\beta {\tilde{T}}_{m}\right)}^{2}+\frac{1}{2-\xi }{{\tilde{u}}_{q}}^{2}+\frac{1}{2b-\xi }{\left(b\delta +\alpha {\tilde{u}}_{d}\right)}^{2})$$

As *ξ* is an adjustable parameter, the extreme values for the denominators of the three terms on the right-hand side are found as follows:

$${\left.\max(\xi (2\sigma -\xi ))\right|}_{\xi =\sigma }=\sigma^{2},\;{\left.\max(\xi (2-\xi ))\right|}_{\xi =1}=1,\;{\left.\max(\xi (2b-\xi ))\right|}_{\xi =b}=b^{2}$$

Consequently,

$$C_{e}(t)\leq \frac{1}{2\sigma^{2}}{\left(\beta {\tilde{T}}_{m}\right)}^{2}+\frac{1}{2}{{\tilde{u}}_{q}}^{2}+\frac{1}{2b^{2}}{\left(b\delta +\alpha {\tilde{u}}_{d}\right)}^{2}+\varepsilon_{e}$$

Here, $\underset{t\to \infty }{\lim}\varepsilon_{e}=0$. Therefore, the supremum ellipsoid of the chaotic attractor is derived as

$$\prod :{y_{1}}^{2}+{y_{2}}^{2}+{y_{3}}^{2}=\frac{1}{\sigma^{2}}{\left(\beta {\tilde{T}}_{m}\right)}^{2}+{{\tilde{u}}_{q}}^{2}+\frac{1}{b^{2}}{\left(b\delta +\alpha {\tilde{u}}_{d}\right)}^{2}$$

**Figure 6:**
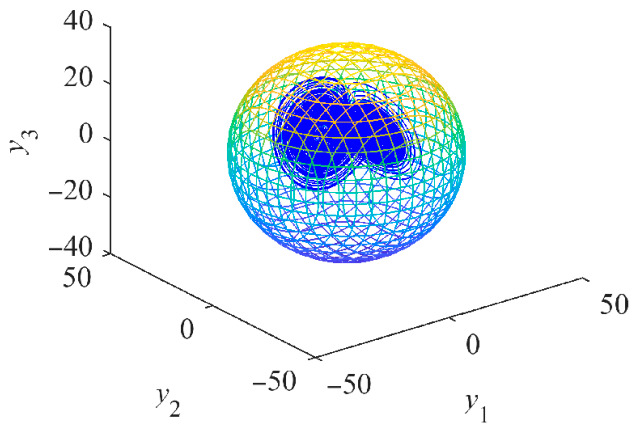
Supremum ellipsoid (parula colormap) of the chaotic attractor (blue color) of system (12) with *u*_d_ = −100 V, *u*_q_ = 0 and *T*_m_ = 0.

The chaotic attractor of system (12) is contained within the supremum ellipsoid ∏, as illustrated in Figure 6. In fact, all trajectories of system (11) are ultimately bounded within the ellipsoid ∏. The supremum surface enables the proper selection of MOSFET current stress ratings for the generator-side three-phase rectifier by defining the limit values of the stator currents. The derived supremum ellipsoid is generally applicable to both salient-pole and non-salient PMSGs, with the parameter values employed here serving as an illustrative example. In the context of turbulent wind conditions, the maximum wind speed is employed to extract the boundary limit values of the stator currents from the supremum surface.

## 4. Chaotic Analysis of the Salient Kolmogorov System

In a salient-pole PMSG, the d-axis and q-axis inductances are unequal, i.e., *L*_d_ ≠ *L*_q_. According to (11), the model of the salient-pole Kolmogorov system is derived as

(22) $$\left(\begin{array}{c} {\dot{y}}_{1}\\ {\dot{y}}_{2}\\ {\dot{y}}_{3} \end{array}\right)=\left(\begin{array}{c} e_{1}y_{2}y_{3}+c_{1}y_{2}\\ e_{2}y_{1}y_{3}-c_{1}y_{1}\\ e_{3}y_{1}y_{2} \end{array}\right)-\left(\begin{array}{c} \sigma y_{1}\\ y_{2}\\ by_{3} \end{array}\right)+\left(\begin{array}{c} \beta {\tilde{T}}_{m}\\ -{\tilde{u}}_{q}\\ -b\delta -\alpha {\tilde{u}}_{d} \end{array}\right)$$

For convenience of comparison, the q-axis inductance of the salient-pole PMSG is taken as *L*_q_ = 12.25 mH and the other operating parameters are the same as in Table 1. By assigning *α* = 0.7, we obtain $\beta =\sqrt{\frac{1-\alpha^{2}}{\varepsilon }}=1.89$.

Firstly, with the dissipative component and external torque removed from (22), the Casimir energy function is chosen as

$$C_{e}=\frac{1}{2}<x,x>=\frac{1}{2}({y_{1}}^{2}+{y_{2}}^{2}+{y_{3}}^{2})$$

Then, the time derivative of *C*_e_ vanishes, indicating a constant Casimir energy determined by the initial condition **x**_0_.

Secondly, regardless of the external torque **f**, the derivative of *C*_e_ can be expressed as

$${\dot{C}}_{e}=-\sigma {y_{1}}^{2}-{y_{2}}^{2}-b{y_{3}}^{2}\leq 0$$

Thus, system (22) is asymptotically stable.

**Figure 7:**
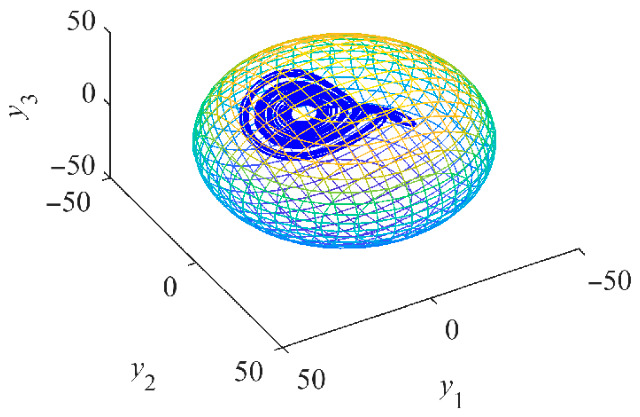
Supremum ellipsoid (parula colormap) of the chaotic attractor (blue color) of system (22) with *u*_d_ = −110 V, *u*_q_ = 10 and *T*_m_ = 2.

Consequently, when considering the combined effects of all terms in (22), the supremum of the resulting chaotic attractor of (22) can be drawn in Figure 7.

## 5. Coexisting Attractors and Bifurcation Analysis

As discussed in Section 2, the non-salient Kolmogorov system (12) possesses three equilibrium points, the stability of which depends on the operating parameters. The Jacobian matrix of (12) is given by

$$J_{\mathrm{ac}}=\left[\begin{array}{ccc} -\sigma & c_{1}+e_{1}{y_{3}}^{*} & e_{1}{y_{2}}^{*}\\ e_{2}{y_{3}}^{*}-c_{1} & -1 & e_{2}{y_{1}}^{*}\\ e_{3}{y_{2}}^{*} & e_{3}{y_{1}}^{*} & -b \end{array}\right]$$

The characteristic polynomial of **J**_ac_ is derived as

(23) $$P=a_{1}\lambda^{3}+a_{2}\lambda^{2}+a_{3}\lambda^{1}+a_{4}$$

here, *a*_1_ = 1, *a*_2_ = *b* + *σ* + 1,

*a*_3_ = *b* + *σ* + *bσ* + *c*_1_^2^ − *e*_1_*e*_2_(*y*_3_^*^)^2^ − *e*_1_*e*_3_(*y*_2_^*^)^2^ − *e*_2_*e*_3_(*y*_1_^*^)^2^ + *c*_1_*e*_1_*y*_3_^*^ − *c*_1_*e*_2_*y*_3_^*^,

*a*_4_ = *bσ* + *bc*_1_^2^ − *e*_1_*e*_3_(*y*_2_^*^)^2^ + *bc*_1_*e*_1_*y*_3_^*^ − *bc*_1_*e*_2_*y*_3_^*^ − *be*_1_*e*_2_(*y*_3_^*^)^2^ − *e*_2_*e*_3_*σ*(*y*_1_^*^)^2^ + *c*_1_*e*_1_*e*_3_*y*_1_^*^y_2_^*^ − *c*_1_*e*_2_*e*_3_*y*_1_^*^y_2_^*^ − 2*e*_1_*e*_2_*e*_3_*y*_1_^*^y_2_^*^y_3_^*^,

$\tilde{Q}=\frac{3a_{3}-{a_{2}}^{2}}{9}$ and $\tilde{E}=\frac{9a_{2}a_{3}-27a_{4}-2{a_{2}}^{3}}{54}$. The eigenvalues of (23) can be derived as

(24) $$\left\{\begin{array}{l} \lambda_{1}=\sqrt[{3}]{\tilde{E}+\sqrt{{\tilde{Q}}^{3}+{\tilde{E}}^{2}}}+\sqrt[{3}]{\tilde{E}-\sqrt{{\tilde{Q}}^{3}+{\tilde{E}}^{2}}}-\frac{1}{3}a_{2}\\ \lambda_{2,3}=-\frac{1}{2}(\sqrt[{3}]{\tilde{E}+\sqrt{{\tilde{Q}}^{3}+{\tilde{E}}^{2}}}+\sqrt[{3}]{\tilde{E}-\sqrt{{\tilde{Q}}^{3}+{\tilde{E}}^{2}}})-\frac{1}{3}a_{2}\\ \;\;\;\;\;\;\pm \frac{1}{2}i\sqrt{3}(\sqrt[{3}]{\tilde{E}+\sqrt{{\tilde{Q}}^{3}+{\tilde{E}}^{2}}}-\sqrt[{3}]{\tilde{E}-\sqrt{{\tilde{Q}}^{3}+{\tilde{E}}^{2}}}) \end{array}\right.$$

**Figure 8:**
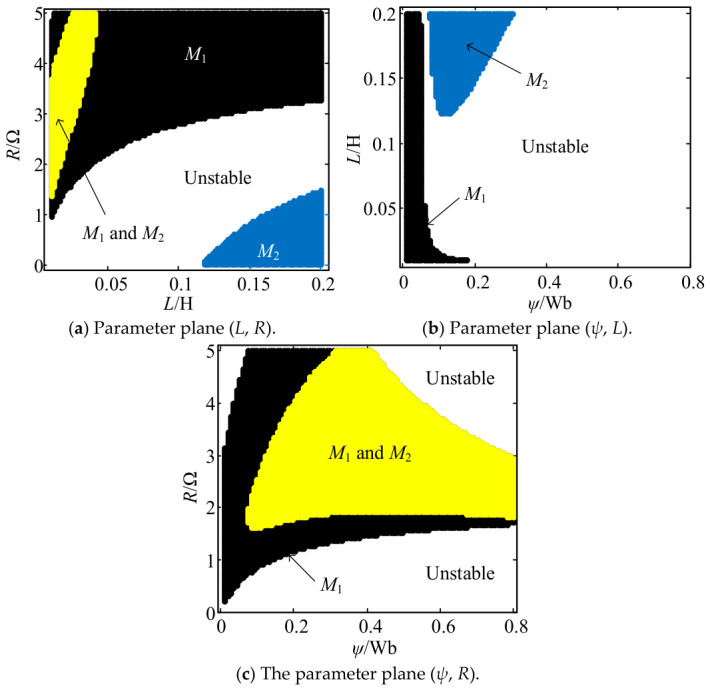
Stability charts of *M*_1_ and *M*_2_ in the parameter planes with *u*_d_ = −200 V, *u*_q_ = −50 V and *T*_m_ = 5 N·m. *M*_1_ and *M*_2_: yellow; *M*_1_: black; *M*_2_: dark blue; Unstable: white.

Through eigenvalue analysis, Figure 8 depicts the stability regions for system (12) derived from parameter sweeps in the (*L*, *R*), (*ψ*, *L*) and (*ψ*, *R*) planes, where the colored areas represent static attractors. The attracting regimes of *M*_1_, *M*_2_ and *M*_3_ confirm the existence of coexisting static attractors with initial conditions initialized at the corresponding equilibria. Stable equilibria *M*_1_ and *M*_2_ coexist under certain conditions, whereas *M*_3_ remains unstable.

**Figure 9:**
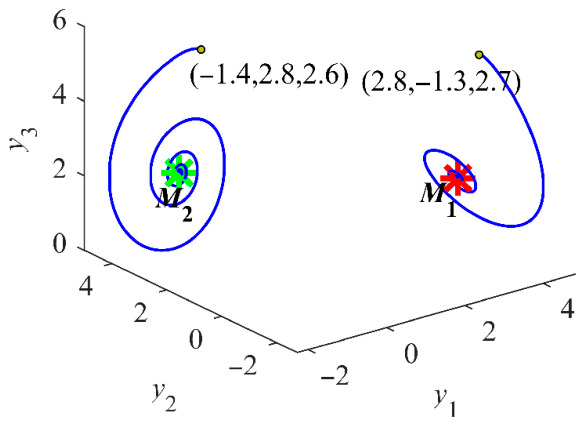
Coexisting stable attractors of the non-salient Kolmogorov system with *L* = 14.25 mH and *R* = 3 Ω. Trajectories: blue; Markers of *M*_1_ and *M*_2_ are depicted in red and green, respectively.

By referring to Figure 8, the design parameters corresponding to the desired rated state are determined. The phase portraits of the stable attractors *M*_1_ and *M*_2_ are plotted in Figure 9. For a PMSG, only one stable equilibrium point corresponding to the rated operating state is necessary, as different equilibrium points are distinctly separated in the phase space. The stability analysis of candidate equilibria provides a theoretical basis for ensuring the system stabilizes at the desired equilibrium point. An effective chaos suppression controller requires that the control input *u*_d_ and *u*_q_ be constrained to stabilize the PMSG at the desired rated equilibrium point while ensuring MOSFET current stress remains within predefined safe operating limits.

**Figure 10:**
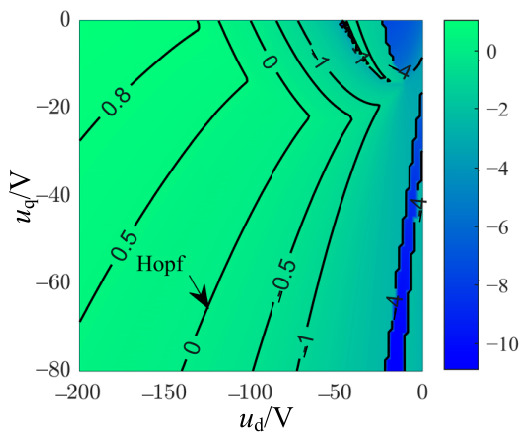
Real parts of the conjugate complex eigenvalues of the non-salient Kolmogorov system with *T*_m_ = 5 N∙m.

**Figure 11:**
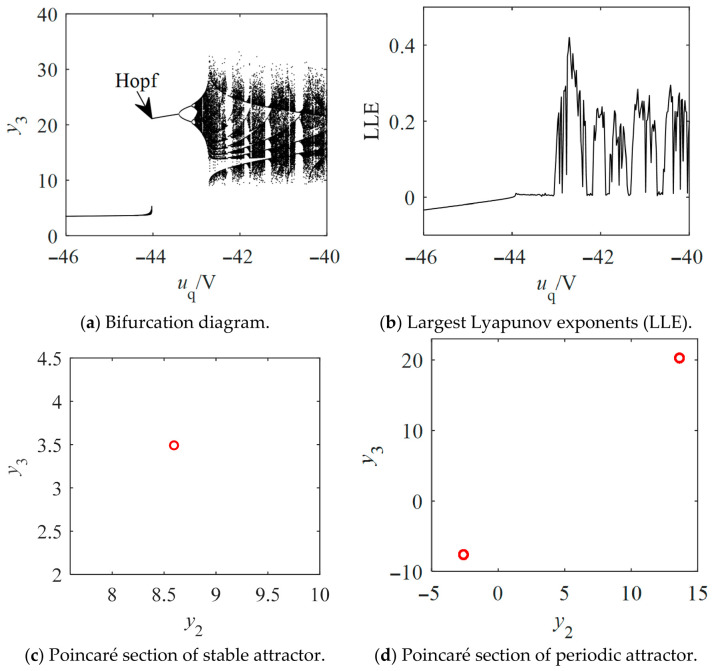
Bifurcation diagrams, LLEs and Poincaré sections of the non-salient Kolmogorov system with *T*_m_ = 5 N∙m, *L*_d_ = *L*_q_ = 14.25 mH and *u*_d_ = −100 V.

To elucidate the route to chaos, a Hopf bifurcation analysis of the equilibrium points is conducted below. Analysis of the Hopf bifurcation conditions at the equilibrium point ${\tilde{M}}_{2}$ confirms that $\frac{d}{d}{Re\left(\lambda \right)|}_{u_{d}=u_{d0,}u_{q}=u_{q0,}}\neq 0$, with λ=±iω and ω≠0. Figure 10 shows the contour plot of the real part of the conjugate complex eigenvalues for the non-salient Kolmogorov system in the parameter region (*u*_d_, *u*_q_). Subsequently, the safe operating regions for *u*_d_ and *u*_q_ could be established. By finding the local maxima of *y*_3_, the bifurcation diagram for the non-salient Kolmogorov system is presented in Figure 11a. The system (12) undergoes a period-doubling bifurcation following the Hopf bifurcation, which ultimately leads to the formation of a chaotic attractor confirmed by the positive largest Lyapunov exponents (LLE) shown in Figure 11b. A comparison with the bifurcation diagram corroborates that as *u*_q_ increases, the largest Lyapunov exponent transitions through negative, zero, and positive regions, corresponding to stable, periodic, and chaotic states, respectively. The chaotic regions are punctuated by periodic windows. With the Poincaré section defined by $\Sigma =\{X={\left(y_{1},y_{2},y_{3}\right)}^{T}\in R^{3}|y_{1}=y_{1}^{*}\}$, Figure 11c,d shows the Poincaré section for the stable attractor at *u*_q_ = −46 V and the Poincaré section for the periodic attractor at *u*_q_ = −43.5 V, respectively. 

**Figure 12:**
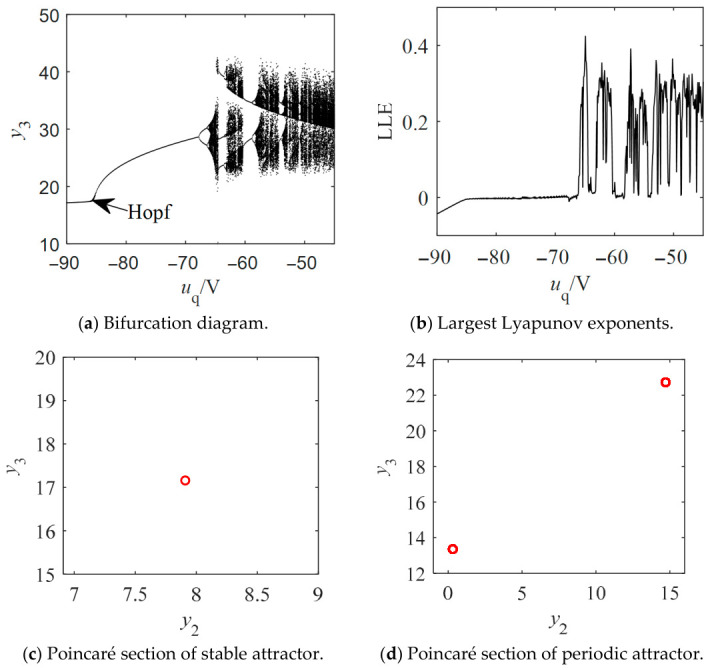
Bifurcation diagrams, LLEs and Poincaré sections of the salient Kolmogorov system with *T*_m_ = 5 N∙m, *L*_d_= 14.25 mH, *L*_q_ = 12.25 mH and *u*_d_ = −100 V.

**Figure 13:**
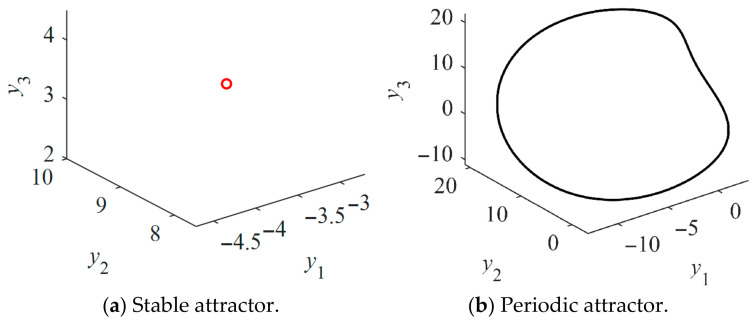
Phase portraits of the non-salient Kolmogorov system.

**Figure 14:**
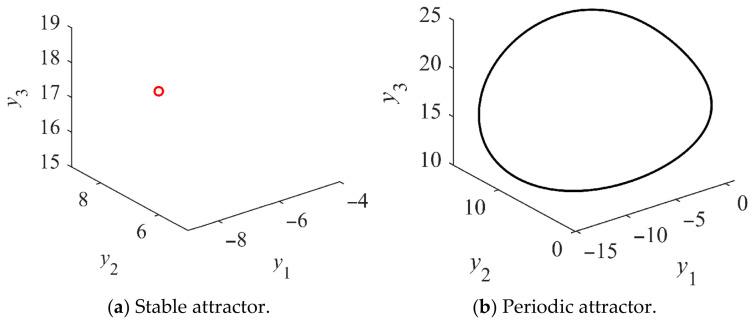
Phase portraits of the salient-pole Kolmogorov system.

Likewise, the bifurcation diagram of the salient-pole Kolmogorov system is depicted in Figure 12a. The salient-pole Kolmogorov system also undergoes both Hopf and period-doubling bifurcations, which ultimately leads to chaos. The largest Lyapunov exponents shown in Figure 12b are used to identify the presence of the chaotic attractor. As corroborated by the bifurcation diagram, the largest Lyapunov exponent transitions from negative to zero and finally to positive values as *u*_q_ increases. These variations correspond to stable, periodic, and chaotic states, respectively, with the chaotic regimes being interspersed with periodic windows. Figure 12c,d shows the stable section at *u*_q_ = −90 V and the periodic section at *u*_q_ = −80 V, respectively. Figure 13a,b shows the stable and periodic attractors of the non-salient Kolmogorov system at steady state. Stable and periodic attractors of the salient-pole Kolmogorov system are shown in Figure 14a,b at steady state.

## 6. Numerical Examples

This section illustrates an example using the previously described analysis method. The chaotic behaviors of the Kolmogorov system are further explored below. For system (12), considering the symmetrical stator voltages, we have *u*_q_ = 0 and *u*_d_ = *U*_m_, where *U*_m_ denotes the amplitude of the three-phase stator voltages in the *abc* reference frame. The equilibrium points of (12) under no-load conditions are given by

(25) $$\left\{\begin{array}{l} {\tilde{M}}_{1}:\left\{0,0,-\alpha {\tilde{u}}_{d}-\delta \right\}\\ {\tilde{M}}_{2}:\left\{-\beta \sqrt{-\gamma -1-{\tilde{u}}_{d}},\sqrt{-\gamma -1-{\tilde{u}}_{d}},\alpha \left(\gamma +1\right)-\delta \right\}\\ {\tilde{M}}_{3}:\left\{\beta \sqrt{-\gamma -1-{\tilde{u}}_{d}},-\sqrt{-\gamma -1-{\tilde{u}}_{d}},\alpha \left(\gamma +1\right)-\delta \right\} \end{array}\right.$$

The eigenvalues of ${\tilde{M}}_{1}$ are obtained as

$$\left\{-1,\frac{1}{2}\left(-1-\sigma \pm \sqrt{{\left(1-\sigma \right)}^{2}-4\sigma (\gamma +{\tilde{u}}_{d})}\right)\right\}$$

when ${\tilde{u}}_{d}\leq -1-\gamma$, ${\tilde{M}}_{1}$ is unstable; otherwise, ${\tilde{M}}_{1}$ is stable. Moreover, ${\tilde{M}}_{2}$ and ${\tilde{M}}_{3}$ exist when ${\tilde{u}}_{d}\leq -1-\gamma$. The variation of ${\tilde{u}}_{d}$ results in the pitchfork bifurcation. A single stable equilibrium point transitions into a pair of stable equilibrium points as ${\tilde{u}}_{d}$ crosses −1−γ. The characteristic polynomial of ${\tilde{M}}_{2}$ and ${\tilde{M}}_{3}$ is expressed as

(26) $${\hat{\lambda }}^{3}+{\hat{a}}_{1}{\hat{\lambda }}^{2}+{\hat{a}}_{2}\hat{\lambda }+{\hat{a}}_{3}=0$$

**Figure 15:**
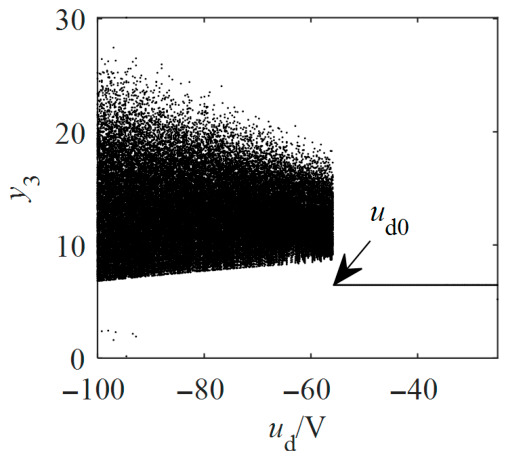
Real part of the rightmost eigenvalues and the bifurcation diagram of (25) with *T*_m_ = 0 and *u*_q_ = 0.

Here, $\hat{\lambda }$ denotes the eigenvalue. ${\hat{a}}_{1}=2+\sigma$, ${\hat{a}}_{2}=-\gamma +\sigma -{\tilde{u}}_{d}$ and ${\hat{a}}_{3}=-2\sigma (1+\gamma +{\tilde{u}}_{d})$. Therefore, the eigenvalues of (26) are analytically determined by (24). The observed symmetry of ${\tilde{M}}_{2}$ and ${\tilde{M}}_{3}$ about the *y*_1_ and *y*_2_ axes suggests a double Hopf bifurcation. By evaluating the double Hopf bifurcation conditions at the equilibrium points ${\tilde{M}}_{2}$ and ${\tilde{M}}_{3}$, it is found that $\frac{d}{d}{Re\left(\lambda \right)|}_{u_{d}=u_{d0}}\neq 0$, with λ=±iω and ω≠0. After that, a chaotic attractor emerges in the Kolmogorov system, as illustrated in Figure 15. Prior to the double Hopf bifurcation, the stable states ${\tilde{M}}_{2}$ and ${\tilde{M}}_{3}$ coexist as two distinct static attractors.

**Figure 16:**
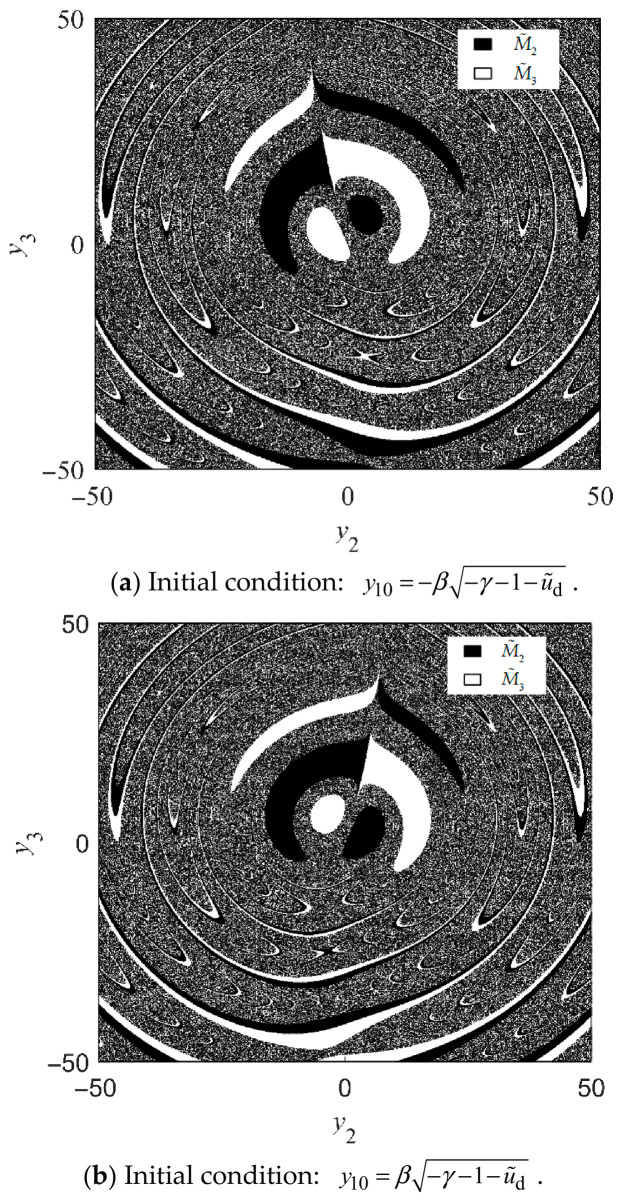
Basins of attraction of ${\tilde{M}}_{2}$ and ${\tilde{M}}_{3}$ with *u*_d_ = −50 V, *T*_m_ = 0 and *u*_q_ = 0.

**Figure 17:**
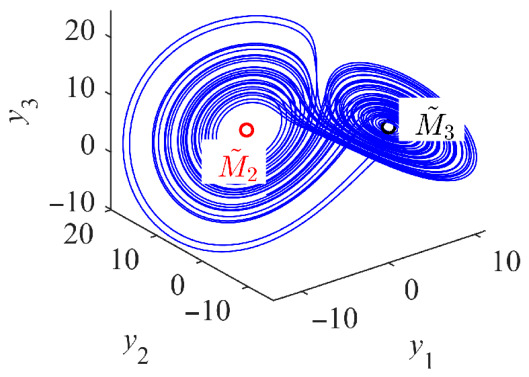
Chaotic attractor of the Kolmogorov system.

For ease of analyzing coexisting attractors, the basins of attraction for the equilibrium point x^*^ are formally defined as B(x^*^) = {*x*_0_|lim*_t_*_→∞_ *φ*(*t*, *x*_0_) = x^*^}, where *x*_0_ denotes the initial value of the variables *y*_1_, *y*_2_ or *y*_3_. Figure 16a,b illustrates the basins of attraction for ${\tilde{M}}_{2}$ (black) and ${\tilde{M}}_{3}$ (white), generated by sweeping the initial conditions of *y*_2_ and *y*_3_ across the interval [−50, 50], when the initial value of *y*_1_ is equal to $-\beta \sqrt{-\gamma -1-{\tilde{u}}_{d}}$ and $\beta \sqrt{-\gamma -1-{\tilde{u}}_{d}}$, respectively. It is observed that the Kolmogorov system converges to distinct equilibrium points depending on the initial conditions. With a continuous decrease in *u*_d_, ${\tilde{M}}_{2}$ and ${\tilde{M}}_{3}$ jointly lose stability and evolve into two unstable vortex centers within the chaotic attractors, as depicted in Figure 17.

In practical applications, it is essential for a PMSG to operate at its rated state, ensuring that stator currents and voltages remain within permissible limits. The emergence of coexisting attractors signifies uncertain steady states that pose a threat to the safe operation of the PMSG. Given that coexisting stable equilibrium points are sensitive to initial conditions, the initial state must be confined to the desired region to avoid converging to abnormal equilibrium points. Within this region, the standard PID controller can still be employed. The conditions governing the emergence of coexisting stable attractors also provide a theoretical basis for their suppression through the appropriate selection of design parameters.

## 7. Conclusions

This article investigates the Kolmogorov model of the salient-pole PMSG, with a discussion on its stability under different terms of the model. The boundary ellipsoid of the chaotic attractor is determined by analyzing the Casimir energy function. Through eigenvalue analysis, the coexistence of the stable attractors and the chaotic butterfly attractor is characterized. Poincaré sections, Lyapunov exponents and phase portraits are utilized to further corroborate the findings of the eigenvalue analysis. Both period-doubling and double Hopf bifurcations induce chaotic behaviors in the Kolmogorov system. Finally, the basins of attraction are mapped, revealing how different initial conditions lead to distinct equilibrium points.

## Figures and Tables

**Table 1:** Operating parameters.

Symbol	Description	Values
*n*_p_	number of pole pairs	1
*B*	Viscous coefficient	0.0162 N/(rad/s)
*L*_d_	d-axis inductance	14.25 mH
*L*_q_	q-axis inductance	14.25 mH
*J*	inertia	4.7 × 10^−5^ kg·m^2^
*ψ*_r_	flux magnitude	0.212 Wb
*R*	resistance	0.9 Ω

## Data Availability

The original contributions presented in the study are included in the article, further inquiries can be directed to the corresponding author.

## Abbreviations

The following abbreviations are used in this manuscript:

PMSM	Permanent-magnet synchronous motors
PMSG	Permanent-magnet synchronous generators

## References

[1] Wu B., Lang Y.Q., Zargari N., Kouro S. (2011). Power Conversion and Control of Wind Energy Systems.

[2] Hemati N., Kwatny H. Bifurcation of equilibria and chaos in permanent-magnet machines. Proceedings of the 32nd IEEE Conference on Decision and Control.

[3] Chau K.T., Wang Z. (2011). Chaos in Electric Drive Systems: Analysis, Control and Application.

[4] Yang G.L., Li H.G. (2009). Sliding mode variable-structure control of chaos in direct-driven permanent magnet synchronous generators for wind turbines. Acta Phys. Sin..

[5] Meng Q., Ren Y., Liu H. (2024). Frequency stability analysis of grid-forming PMSG based on virtual synchronous control. IEEE Access.

[6] Yang J., Yan H., Gu C., Wang S., Zhao W., Wheeler P. (2022). Modeling and stability enhancement of a permanent magnet synchronous generator based dc system for more electric aircraft. IEEE Trans. Ind. Electron..

[7] Huang J., Zhang Z., Han J. (2022). Stability analysis of permanent magnet generator system with load current compensating method. IEEE Trans. Smart Grid.

[8] Kim Y.W., Sul S.K. (2023). Stability analysis of active front end and permanent magnet synchronous generator with back emf-based sensorless control for dc marine vessels. IEEE Trans. Power Electr..

[9] Chen W., Kou W., Wei Z., Wang B., Li Q. (2024). Analysis of bifurcation characteristics of fractional-order direct drive permanent magnet synchronous generator. Energy Sci. Eng..

[10] Li L., Xiao L., Zuo Q., Tan P., Wang Y. (2025). A novel neural dynamics controller for weakening the chaos of permanent magnet synchronous generator and its extended application. IEEE Trans. Cybern..

[11] Suyapan A., Areerak K., Bozhko S., Yeoh S., Areerak K. (2021). Adaptive stabilization of a permanent magnet synchronous generator-based dc electrical power system in more electric aircraft. IEEE Trans. Transp. Electrif..

[12] Zhang C., Plestan F. (2021). Adaptive sliding mode control of floating offshore wind turbine equipped by permanent magnet synchronous generator. Wind Energy.

[13] Babaghorbani B., Beheshti M.T., Talebi H.A. (2021). A Lyapunov-based model predictive control strategy in a permanent magnet synchronous generator wind turbine. Int. J. Electr. Power.

[14] Luo S., Song Y., Lewis F.L., Garrappa R., Li S. (2023). Dynamic analysis and fuzzy fixed-time optimal synchronization control of unidirectionally coupled of permanent magnet synchronous generator system. IEEE Trans. Fuzzy Syst..

[15] Zhu D., Wang R., Liu C., Duan J. (2020). Synchronization of chaotic-oscillation permanent magnet synchronous generators networks via adaptive impulsive control. IEEE Trans. Circuits Syst. II.

[16] Qi G., Zhang J. (2017). Energy cycle and bound of Qi chaotic system. Chaos Soliton Fract..

[17] Qi G. (2017). Energy cycle of brushless DC motor chaotic system. Chaos Soliton Fract..

[18] Jia H., Shi W., Wang L., Qi G. (2020). Energy analysis of Sprott-A system and generation of a new Hamiltonian conservative chaotic system with coexisting hidden attractors. Chaos Soliton Fract..

[19] Ji’e M., Yan D., Sun S., Zhang F., Duan S., Wang L. (2021). A Simple Method for Constructing a Family of Hamiltonian Conservative Chaotic Systems. IEEE Trans. Circuits Syst. I.

[20] Zhang Z., Huang L. (2022). A new 5D Hamiltonian conservative hyperchaotic system with four center type equilibrium points, wide range and coexisting hyperchaotic orbits. Nonlinear Dyn..

[21] Liu T., Yan H., Banerjee S., Mou J. (2021). A fractional-order chaotic system with hidden attractor and self-excited attractor and its DSP implementation. Chaos Soliton Fract..

[22] Li Q., Chen W., Wei Z., Wang K., Wang B. (2024). Hopf bifurcation analytical expression and control strategy in direct-drive permanent magnet synchronous generator. Int. J. Circ. Theor. Appl..

[23] Tahmasbi M. (2024). Chaos control in networked permanent magnet synchronous motor using Lyapunov-based model predictive subject to data loss. Eng. Rep..

[24] Spiegel M.R., Lipschutz S., Liu J. (2009). Mathematical Handbook of Formulas and Tables.

